# Isolation, characterization and optimization of indigenous acetic acid bacteria and evaluation of their preservation methods

**Published:** 2010-03

**Authors:** SM Sharafi, I Rasooli, K Beheshti-Maal

**Affiliations:** 1 M.Sc (Microbiology student), Department of Biology, Shahed University, Tehran-Qom Express Way, Opposite Imam Khomeini's shrine, Tehran-3319118651, Iran; 2 Islamic Azad University, Falavarjan-Iran

**Keywords:** Acetic acid bacteria, *Acetobacter pasteurianus*, Vinegar, Isolation, Iran

## Abstract

**Background and Objectives:**

Acetic acid bacteria (AAB) are useful in industrial production of vinegar. The present study aims at isolation and identification of acetic acid bacteria with characterization, optimization, and evaluation of their acetic acid productivity.

**Materials and Methods:**

Samples from various fruits were screened for presence of acetic acid bacteria on glucose, yeast extract, calcium carbonate (GYC) medium. Carr medium supplemented with bromocresol green was used for distinguishing *Acetobacter* from *Gluconobacter*. The isolates were cultured in basal medium to find the highest acetic acid producer. Biochemical tests followed by 16S rRNA and restriction analyses were employed for identification of the isolate and phylogenic tree was constructed. Bacterial growth and acid production conditions were optimized based on optimal inoculum size, pH, temperature, agitation, aeration and medium composition.

**Results:**

Thirty-seven acetic acid bacteria from acetobacter and gluconobacter members were isolated. Acetic acid productivity yielded 4 isolates that produced higher amounts of acid. The highest producer of acid (10.03%) was selected for identification. The sequencing and restriction analyses of 16S rRNA revealed a divergent strain of *Acetobacter pasteurianus* (Gene bank accession number#GU059865). The optimum condition for acid production was a medium composed of 2% glucose, 2% yeast extract, 3% ethanol and 3% acid acetic at inoculum size of 4% at 3L/Min aeration level in the production medium. The isolate was best preserved in GYC medium at 12°C for more than a month. Longer preservation was possible at −70°C.

**Conclusion:**

The results are suggestive of isolation of an indigenous acetic acid bacteria. Pilot plan is suggested to study applicability of the isolated strain in acetic acid production.

## INTRODUCTION

Acetic acid bacteria are a large group of obligate aerobic gram negative bacteria with the ability to oxidize ethanol to acetic acid (1). They are widely distributed in natural habitats and classified into the family *Acetobacteraceae*. Members of this family are useful in industrial production of vinegar (2). Acetic acid bacteria (AAB) can use substrates such as glucose, ethanol, lactate or glycerol as energy sources. However, most of these compounds are not completely oxidized into CO_2_, and water and several metabolites, especially acetic acid, are accumulated in the growth medium. AAB are commonly found in nature because of their high resistance to acidity and the variety of substrates that they can use (3). These bacteria have been isolated from alcoholic beverages, vinegar, fruits, flowers, honey, bees, sugar cane juices, soil, and water (4). Isolation of AAB with high production and better acidotolerance potentials has been subject of research. The thermotolerant strains were able to oxidize ethanol at high temperatures (38–40°C) and ethanol concentrations (up to 9%) without any appreciable lag time; they worked rapidly with a higher fermentation rate, where mesophilic strains were unable to do this. Recently, Ndoye *et al* 2006 (5) have selected two strains of *A. tropicalis* and *A. pasteurianus* species for their ability to grow at 40 and 45°C and proposed them to produce an artisanal spirit vinegar.

This study aims to isolate and identify acetic acid bacteria from fruits, characterize and optimize indigenous acetic acid bacteria and evaluate their productivity and to compare their growth characteristics. Since Isfahan is located in a tropical area, it is likely that species of acetic acid bacteria with good acid production potentials will be found in this part of Iran. Identification of the isolated acetic acid bacterium was carried out by 16S rRNA analysis.

## MATERIALS AND METHODS

**Materials.** Chemical reagents and culture media were from Sigma (USA) or Merck (country). Restriction endonucleases were obtained from Cinagene (Iran). Gel purification kit was from Bioneer (Korea). Primers were synthesized by Bioneer.

**Primary screening.** These isolates were from apple, fig, grape, apricot, date, nectarine, sloe, peach, pear and flame. Ripened samples were collected in August–September 2008. They were then left for a few days for over-ripening and were subsequently crushed aseptically and were bottle incubated at 30°C for 7 days. 100 µl from different dilutions of the bottles were then spread on plates of glucose solid GYC medium (10% glucose, 1.0% yeast extract, 2.0% calcium carbonate, 1.5% agar, pH 6.8) supplemented with 100 mg l^−1^ of Pimaricin (Sigma-Aldrich; Steinheim; Germany) to inhibit the growth of yeasts and moulds. This antibiotic was added to the culture medium from the stock solution after the medium had been sterilized. Plates were incubated at 30°C for 3–4 days under aerobic conditions. Colonies showing a clear halo on GYC were selected to isolate dominant species with high probability (6). Acetobacter and Gluconobacter are distinguished from the Acetobacteriacea family on the basis of acid production from calcium carbonate. Morphological and cultural characteristics of the isolates were examined by incubation at 30°C for 2 days on GYC medium. Acetobacter and Gluconobacter were distinguished from each other on Carr medium in the presence of bromocresol green. Acetobacter turns the media color to yellow and then to green while Gluconobacter turns it into yellow.

**Acetic acid production.** The selected colonies from GYC were transferred to Brain Heart Infusion (BHI) broth until OD_600_ of 0.5 was achieved. Inoculums size of 4% from the preceding BHI broth was cultured in Yeast Glucose ethanol acetic acid (YGEA) medium for acetic acid production. The production medium was aerated and samples were taken at 48 hour intervals.

**Estimation of acetic acid.** 5ml of the culture was mixed with 20ml of distilled water. 3–5 drops of phenolphthalein indicator was added. The solution was titrated against 0.5N NaOH. The amount (g) of acetic acid produced in 100 ml of medium was calculated using the following formula:

Acetic acid(g/100ml)=Volume of NaOH(ml)used in titration×0.03×20

**Identification of *Acetobacter* spp.** The following tests were performed to identify the Acetobacter Spp. isolated. Catalase, production of acid from D-glucose, nitrate reduction, production from D-glucose of 5-keto-D-gloconate and Ketogenesis from glycerol (7, 8). Nitrate reduction was tested from nitrate peptone water (per liter of distilled water, pH 7.0: peptone, 10 g; KNO_3_, 2 g) (9). The biochemical identification tests were followed by molecular methods to validate the data obtained thereby.

**Identification by sequencing of 16S rRNA gene.** The bacteria were grown in GYC medium for 2–5 days at 30°C. The chromosomal DNA was extracted as described by Sambrook et al (10).

**PCR amplification and analysis of the products.** Primers for the PCR amplifcation of 16S rRNA were selected from conserved regions of the 5′- end (16Sd, 5′-′ AGAGTTTGATCCTGGCTCAG -3′) and the 3′-end (16Sr, 5′- ACGGCTACCTTGTTACGACCT-3′) of the 16S rRNA. The PCR products (1,500 bp for 16S rRNA) were purified using a PCR purification kit as per the manufacturer's instructions.

AAB isolated strain was identified by sequencing partial 16S rDNA regions. The following primers were used for sequencing:

upper primer: 5′ AGAGTTTGATCCTGGCTCAG 3′

lower primer: 5′ ACGGCTACCTTGTTACGACCT 3′

**Restriction analysis.** PCR product was digested with *Eco*R1 and *Xba*1. Restriction fragments generated by *Eco*R1 and *Xba*1were detected by 2% agarose gel electrophoresis. Lengths of both amplification products and restriction fragments were detected by comparison with DNA ladder. Eight microliters (approximately 50–100 ng DNA) of each PCR amplified 16S rRNA gene from bacterial isolate was digested for 3 h at 37°C with *Eco*R1 and *Xba*1 restriction endonucleases, as recommended by the manufacturer (Invitrogen, Carlsbad, CA, USA). Restriction fragments were analyzed by 2% w/v agarose gel electrophoresis. The isolated bacterial sequence was subjected to software analysis (www.ebi.ac.uk and http://itol.embl.de/) to draw phylogenic tree.

**Optimization of bacterial growth and acetic acid production conditions.** Three factors of culture medium composition, temperature and shaking conditions were considered for culture conditions. Two hundred µl of stock culture (0D_600_=2.3) was inoculated into 15 ml of each of BHI and LB broth. The broth cultures were then incubated at 25, 31, 37°C at two shaking rates of 210 rpm and 180 rpm. The optical density and cell dry weight were measured after 3, 6, 9 and 12 hours. In brief, 5ml of culture medium was centrifuged and the pellet was dried at 60°C for 1 hour and then at 50°C overnight. Three factors of aeration (3L/Min and 2.5L/Min), inoculum size (3 and 4% from BHI broth at OD_600_=0.5), glucose content, yeast extract, ethanol, and acetic acid were considered for production conditions. The basal medium (YGEA) was composed of ethanol, yeast extract, glucose and acetic acid each at 2% level. 200 µl of stock (0D=2.3) was inoculated in 15 ml of BHI broth. An inoculum size of 4% from BHI broth at OD_600_=0.5 was inoculated in production medium under 3L/Min aeration.

**Preservation conditions.** The isolate was preserved in LB, BHI, Nutrient, GYC and Carr media to evaluate the viability, longevity and acid productivity after lapse of at least one month.

## RESULTS

**Fig. 1:**
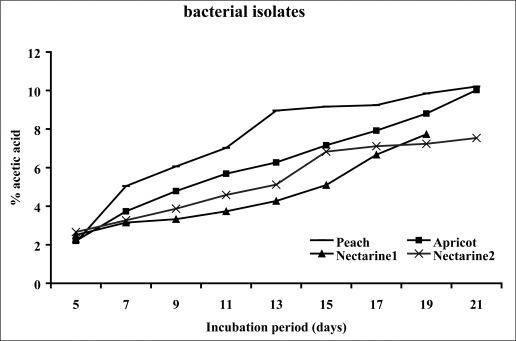
Acetic acid production (g/100ml) in basal medium by bacterial isolates

**Fig. 2:**
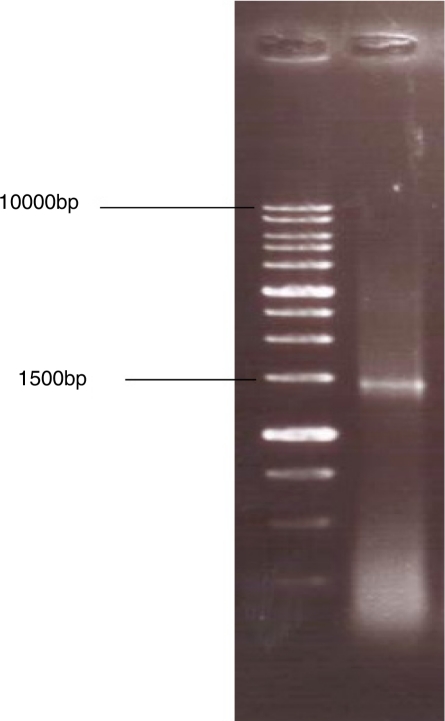
PCR product of the amplified 16s rRNA from the isolated Acetobacter species.

**Fig. 3:**
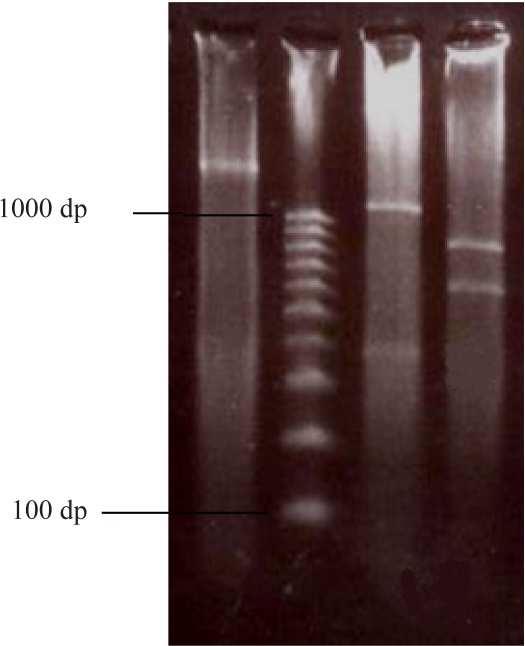
Restriction enzyme digestion of the 16S rRNA gene by *Xba*I and *Eco*RI of the gene fragment from the isolated *Acetobacter* spp. Lane 1=PCR product (1500bp), Lane 2=DNA ladder(100–1000), Lane 3 *Xba*I digested fragment (1220+330bp), Lane 4=digested by *Eco*R1 (925+625).

**Fig. 4:**
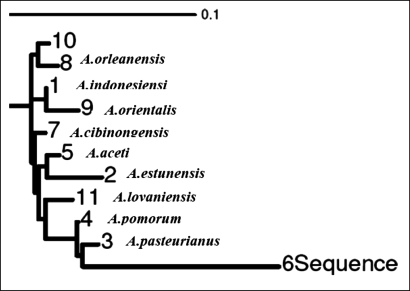
Phylogenetic relationships of Acetobacter strains based on the 16S rRNA sequence.

All strains tested produced acid from D-glucose and ethanol. The strains showed clear zones on basal agar plates containing CaCO_3_. Therefore, they were regarded as *Acetobacter* and *Gluconobacter* and used for further study. A total of 37 isolates were selected as acetic acid bacteria. They were all Gram-negative, circular or rod-shaped aerobes with non-pigmented colonies. The isolates were tested for acetic acid production in the basal medium. Oxidation of acetate to carbon dioxide and water was found positive in 4 samples which were then regarded as Acetobacter. The isolates producing higher amounts of acetic acid were selected (Fig. 1) for further processing until one isolate which produced the highest amount of acetic acid was selected. The bacterial isolate from peach showed the highest acid productivity of 10.03% (Fig. 1). The isolate was negative for oxidase, gelatinase, H_2_S, and indole. The preliminary identification on the basis of biochemical tests (Table 1) brought about the possibility of having one of the three acetobacteria (*A.orleanensis*, *A.pasteurianus and A.pomorum)*. Hence molecular techniques were employed for more precise identification. The 16S rRNA gene was successfully amplified by PCR (Fig. 2). The sequencing analysis of the PCR product by Macrogen company (South Korea) revealed a divergent strain of *A. pasteurianus* (Gene bank accession number # GU059865). The isolated strain exhibited more than 80% sequence similarity based on 16S rRNA sequence analysis (Gene bank accession number # GU059865). Software analysis suggested two restriction sites on the gene. The PCR product was accordingly subjected to *Eco*RI and *Xba*I digestions to obtain 925+625 and 1220+330 fragments respectively (Fig. 3). The phylogenic tree (Fig. 4) shows the highest similarity and closer relationship of the isolate to *A. pasteurianus*. The optimum conditions for bacterial growth were 31oC at a shaking speed of 210 rpm (Table 2). The optimum condition for acid production was a medium composed of 2% glucose, 2% yeast extract, 3% ethanol and 3% acid acetic (Table 3). The isolate also grew well in BHI broth. Inoculum size of 4% from growth medium with 3L/Min aeration level in the production medium was found to be a more efficient production condition.

**Table 1:** Characteristics of the species of the genus *Acetobactera.*

	*Bacterial isolate*	*A.aceti*	*A.cibinongensis*	*A.estunensis*	*A.indonesiensis*	*A.lovaniensis*	*A.orientalis*	*A.orleanensis*	*A.pasteurianus*	*A.pomorum*	*A.sizigii*	*A.tropicalis*
**Catalase**	**+**	**+**	**+**	**+**	**+**	**+**	**+**	**+**	**(+)**	**+**	**+**	**+**
**Ketogenesis from glycerol**	**+**	**+**	–	–	–	–	–	**d**	**d**	**+**	–	–
**Production of acid from D-glucose**	**+**	**+**	**+**	**+**	**+**	**+**	**+**	**+**	**d**	**(+)**	**+**	**+**
**Production from D-glucose of: 5-keto-D-gloconate**	–	**+**	–	–	–	–	–	–	–	–	–	–
**Nitrate reduction**	**+**		–	–	**d**	**d**	–	–	**+**	**nd**	–	–

Symbols:+, 90% or more of the strains positive; (+), weakly positive reaction; d, 11–89% of the strains positive;-, 90% or more of the strains negative; nd, not determined.

**Table 2:** The effect of temperature and agitation on bacterial growth.

Temperature (°C)	Time (h)	180 rpm		210 rpm	

		OD_600_	Bacterial dry weight (mg/ml)	OD_600_	Bacterial dry weight (mg/ml)
	3	0.860	5.8	0.890	6
**25**	6	1.759	7.2	1.80	7.6
	9	1.920	7.8	1.960	8.2
	12	2.010	8.4	2.20	8.8
	3	0.910	7.2	0.98	7.6
**31**	6	1.950	7.6	1.98	8.2
	9	2.210	8.2	2.26	8.8
	12	2.320	9	2.38	9.6
**37**	3	0.660	5	0.70	5.4
	6	1.650	6	1.70	7
	9	1.760	7	1.81	7.8
	12	1.950	7.4	2.10	8.4

**Table 3:** The effect of medium composition on production of acetic acid by the bacterial isolate

Incubation period (days)	1%				2%				3%				4%			

	G	Y	E	A	G	Y	E	A	G	Y	E	A	G	Y	E	A
5	2.22	2.4	2.04	2.04	2.4	2.8	2.1	2.1	2.22	2.1	2.4	2.4	1.98	1.98	2.6	2.6
7	3.6	3.9	3.5	2.8	4.5	4.9	2.54	2.5	3.5	3.5	4.8	3.8	3.3	3.4	3.8	2.8
9	4.4	4.3	5.2	3.1	5.2	5.5	4.4	3.4	4.1	4.2	5.4	4.4	4	4	4.4	3.1
11	4.8	4.5	6.4	4.2	6.6	6.8	5.8	4.8	4.5	4.5	8.5	6.5	4.2	4.2	5.2	4.2

**G**= Glucose, **Y**= yeast extract, **E**= ethanol, **A** =acid acetic

## DISCUSSION

**Isolation and identification of bacterial species.** Overoxidation is a serious problem during vinegar making without temperature control in tropical and temperate countries. Changes in the population or in the physiology of strains, as a result of the lack of alcoholic substrate are reasons for overoxidation (7). New microbial isolates are always needed to meet the biotechnologists’ requirements. The probability to isolate different species from the samples increases irrespective to their relative presence (11). The number of strains able to grow decreases with increase of glucose due to a strong effect of bacterial growth on glucose concentration. In the present study, the isolated bacteria were able to grow at 10% sugar concentration of GYC. Production of 10.03% acetic acid at the initial stage of isolation was promising (Fig. 1). We therefore attempted to characterize the isolates biochemically (Table 1). In addition to their ability to oxidise ethanol, *Acetobacter* and *Gluconacetobacter* species can further oxidise acetic acid to CO_2_ and H_2_O, generating the so-called acetate overoxidation, that is carried out by the tricarboxylic acid cycle when there is a high level of dissolved oxygen and no ethanol in the medium. Strains of *Gluconobacter* are not able to overoxidise because of nonfunctional α-ketoglutarate dehydrogenase and succinate dehydrogenase of tricarboxylic acid cycle; they can only oxidize ethanol to acetic acid (12). Acetic acid bacteria are characterized by the ability to oxidize alcohols or sugars incompletely, and a common feature to most of them is the ability to oxidize ethanol to acetic acid. Acid production from ethanol, generally shown with the method described by Frateur (13) as a clearing of the opacity in the medium around the bacterial growth or with the method described by Carr (14) as a color change of the indicator bromocresol green in the medium from green to yellow (15) confirm our findings in the present study that the isolate is acetobacter. This is further validated by oxidation of acetate to CO_2_ and H_2_O (15) and ketogenesis from glycerol (14) (Table 1). The isolate grew well in the presence of 3% acetic acid. This finding is in support of acetobacter growth in the presence of 0.35% acetic acid (pH 3.5) (16). The majority of isolated strains were able to grow at 7% v/v of ethanol, and some of them at 11% of ethanol. These findings are comparable to those of Gullo *et al.* (17) who reported isolation of bacterial strains with ability to grow at 5% v/v of ethanol. Phenotypic characterization methods of AAB are not reliable and very time-consuming. They have been therefore different molecular techniques have been employed, in particular, DNA:DNA hybridizations (8, 16, 18, 19) and PCR-based genomic fingerprinting techniques such as Restriction Fragment Length Polymorphism (RFLP) analysis of PCR-amplified 16S rRNA (20–23) or 16S– 23S rRNA intergenic spacer regions (21, 23–26), and Randomly Amplified Polymorphic DNA (RAPD) fingerprinting (27, 28). Routine analysis of large amounts of samples that can be isolated from nature is not possible by these molecular methods and Some quick and reliable techniques such as RFLP analysis of PCR-amplified 16S rRNA gene have been considered as appropriate techniques for the differentiation and characterization of microorganisms (29). The accurate identification of this isolate required genotypic characterization because phenotypic characteristics are very similar (17). PCR–RFLP of the 16S rRNA gene has already been used to identify AAB isolates and to characterize reference strains (22, 23). Amplified products of the 16S rRNA gene contained approximately 1500 bp (Fig. 2). This fragment was sequenced (1339bp) and aligned with various acetobacter species. The highest similarity was found with *A. pasteurianus* (. Gene bank accession number # GU059865). PCR–RFLP of the 16S rDNA allowing the identification of the AAB in a shorter period of time, compared with the time-consuming techniques. The use of these techniques for AAB differentiation is proposed for routine laboratory analysis because of its ease, the general use of a single PCR and limited restriction analysis, and for the low cost as compared to the techniques used to identify the novel AAB species. Two restriction endonucleases (*Eco*RI and *Xba*I) were identified on the isolated gene using CLC Sequence Viewer 4. Several authors reported molecular procedures based on the restriction fragment analysis of 16S (22–24). Fig. 3shows the patterns obtained with each of the restriction endonucleases of the 16S rRNA gene needed to identify the isolated bacterium. This analysis confirmed identity of the isolate as *A. pasteurianus*.

Temperature optimization is essential for any biotechnological process as bacterial deactivation processes can occur above optimum temperature. This deactivation is attributed to the essential enzyme denaturation, membrane damage that causes cellular constituent scattering and the organism becoming more sensitive to the toxic effect of acetic acid (30). Minimum and maximum growth temperatures are more difficult to define both for the variability among the species and for the influence of medium composition. About upper temperature limits, several studies have shown the occurrence of thermo-tolerant AAB strains in industrial vinegar production (31). In the present study, the optimum growth temperature was found to be 31°C (Tables 2 – 3).

The optimal pH growth of AAB is between 5.0–6.5 (32). In the present study, the optimum pH for growth and acid production had a wider range of 3–5. AAB are also able to grow at lower pH values where bacterial activity has been detected for pH values < 3 (33). However, the tolerance to low pH is strongly dependent on other parameters such as ethanol concentration and oxygen availability. In conclusion, the results suggest isolation of an indigenous acetic acid bacterium. Pilot plan is suggested to study applicability of the isolated strain in acetic acid production.
